# Analyzing the effectiveness of vocal features in early telediagnosis of Parkinson's disease

**DOI:** 10.1371/journal.pone.0182428

**Published:** 2017-08-09

**Authors:** Betul Erdogdu Sakar, Gorkem Serbes, C. Okan Sakar

**Affiliations:** 1 Department of Software Engineering, Bahcesehir University, Istanbul, Turkey; 2 Department of Biomedical Engineering, Yildiz Technical University, Istanbul, Turkey; 3 Department of Computer Engineering, Bahcesehir University, Istanbul, Turkey; Shanghai Maritime University, CHINA

## Abstract

The recently proposed Parkinson’s Disease (PD) telediagnosis systems based on detecting dysphonia achieve very high classification rates in discriminating healthy subjects from PD patients. However, in these studies the data used to construct the classification model contain the speech recordings of both early and late PD patients with different severities of speech impairments resulting in unrealistic results. In a more realistic scenario, an early telediagnosis system is expected to be used in suspicious cases by healthy subjects or early PD patients with mild speech impairment. In this paper, considering the critical importance of early diagnosis in the treatment of the disease, we evaluate the ability of vocal features in early telediagnosis of Parkinson's Disease (PD) using machine learning techniques with a two-step approach. In the first step, using only patient data, we aim to determine the patient group with relatively greater severity of speech impairments using Unified Parkinson’s Disease Rating Scale (UPDRS) score as an index of disease progression. For this purpose, we use three supervised and two unsupervised learning techniques. In the second step, we exclude the samples of this group of patients from the dataset, create a new dataset consisting of the samples of PD patients having less severity of speech impairments and healthy subjects, and use three classifiers with various settings to address this binary classification problem. In this classification problem, the highest accuracy of 96.4% and Matthew’s Correlation Coefficient of 0.77 is obtained using support vector machines with third-degree polynomial kernel showing that vocal features can be used to build a decision support system for early telediagnosis of PD.

## Introduction

Parkinson’s disease (PD) is one of the most frequently seen neurodegenerative disorders affecting the human central, peripheral, and enteric nervous systems [1]. In a recent study that synthesized studies on the prevalence of PD, meta-analysis of the worldwide data showed that PD prevalence increases steadily with age from 41/100000 in 40 to 49 years to 1903/100000 in older than 80 years [2]. The standardized incidences reported in previous studies ranged from 16 to 19 per 100000 per year [3]. Many studies have reported that PD incidence also rises steadily with age to a peak occurring at the age of 70 to 79 years [4]. However, it is also noted that this may be because of the difficulty in identifying very elderly patients [3]. These findings show that aging of general population will bring about a dramatic increase in in the number of people diagnosed with PD [5]. Therefore, there is an increasing need to build reliable telemedicine systems that alleviates the burden of frequent physical visits to the clinic and uncompensated medical expenditures [6–9]. Although there is no cure for the disease, some symptoms of the Parkinson’s disease can be suppressed by pharmacological or surgical intervention [10]. Thus, early diagnosis is of critical importance as it enables the early introduction of treatment that can improve the quality of life and extend the life span of the patients.

Unified Parkinson's Disease Rating Scale (UPDRS) is the most commonly used rating tool to follow the PD progression and evaluate the results of surgical, medical, and other interventions of the disease [11–13]. The UPDRS is composed of three main components: first is the “mentation, behavior and mood”, which consists of 4 sections; second is the “activities of daily living” which consists of 13 sections and assesses whether a PD patient can fulfill daily tasks without any assist; and the third is “Motor” which consists 27 sections and evaluates muscular control [14, 15]. The effect of speech shows up in two components: primarily in the 5^th^ section of component 2 for assessing whether the patient’s vocal output is apprehensible and secondly in the 18^th^ section of component 3 for evaluating whether the patient’s vocal output is expressive during a conversation. The UPDRS is highly used due to its various strengths: (i) it assesses both motor disability (second component) and motor impairment (third component), (ii) a teaching-videotape is used to standardize the practical application and this enhances the inter-rater reliability [10, 16] (iii) its reliability and validity has been assessed many times due to its clinometric scale evaluation ability. Its reliability was examined in literature in terms of internal consistency [8, 11, 17, 18], inter-rater reliability 11, 17], intra-rater reliability [19], test–retest reliability in elderly patients with parkinsonian signs (but not necessarily PD) [19] and test–retest reliability in patients with early, mild PD [12]. The results have demonstrated that although there are some items focused on the same aspect of the construct, UPDRS is one of the most valid and reliable scale that can be used to follow the course of PD. However, as the patients live longer, even some of the symptoms are treated; it is becoming more and more difficult for the patients to come to visits in hospital for diagnosis, monitoring, and treatment. Therefore, tele-monitoring of signs can complement traditional clinical examinations [20] and decrease the number of physical visits to clinics. Consequently, the life of PD patients and their relatives may be easier and the workload of clinicians may reduce.

Vocal impairment is one of the most important signs of PD since it is seen in approximately 90% of the patients in the earlier stages of the disease [8, 18]. Therefore, there is an increasing interest in building PD diagnosis and telemonitoring systems based on vocal features. The tele-diagnosis systems aim to discriminate PD patients from healthy subjects [8, 21–25] and the telemonitoring systems aim to predict the clinical evaluation metrics in order to track the sign progression of the disease [8, 14, 26]. Most of the telediagnosis studies use an online available Parkinson voice dataset which consists of 195 voice recordings belonging to 23 PD patients and 8 healthy subjects [8]. In a recent study, clustering based feature weighting and complex valued artificial neural network were combined to discriminate healthy subjects from PD patients and 99.52% classification accuracy was achieved on this dataset [22]. Similar studies that address the telediagnosis problem have obtained similar classification performances by combining feature selection and classification algorithms [21, 24, 25].

The PD tele-monitoring studies based on speech recordings of PD patients aim to map the vocal features to a clinical evaluation system used to describe how the signs of Parkinson's disease progress. Since UPDRS is the most widely used scale, many researches are trying to estimate the whole or a part of the UPDRS score using data that is retrieved by teleprocessing. In a study conducted at University of California, data collected with an at-home testing device recording both motor and speech data was used with linear regression to estimate UPDRS score [20]. Khan et. al [27] extracted 13 features, including the cepstral separation difference and Mel-frequency cepstral coefficients, from 240 running speech samples recorded from 60 PD 20 healthy controls and predicted UPDRS Motor Examination of Speech with 85% accuracy by using SVM. In another recent study, Bayestehtashk et al. [14] has collected a relatively large cohort of 168 subjects remotely from three different clinics and obtained mean absolute error of 5.5 in predicting the UPDRS score.

As mentioned above, although there have been many studies aiming at building PD telediagnosis and telemonitoring systems based on vocal features, the ability of vocal features in early telediagnosis of PD have not been investigated yet in the literature. Many literature studies that propose telediagnosis systems based on speech disorders reported very high classification rates in discriminating healthy subjects from PD patients [21–24]. However, in these studies the data used to build the classification model contain the speech recordings of both early and late PD patients with different severities of speech impairments. In a more realistic scenario, the telediagnosis system is expected to be used in suspicious cases by healthy subjects and patients with mild motor system disorders. In the literature, it has been found that speech disorders have the potential to be the early indicators of PD. In [28], using disease duration as an index of disease progression, the association between disease duration and various UPDRS subscores is examined, and the findings revealed that activities of daily living (ADL) subscore and motor subscore, each including a speech part, are strongly associated with disease duration. In this paper, considering the critical importance of early diagnosis in the treatment of the disease, we investigate the usefulness of vocal features in early telediagnosis of Parkinson's Disease (PD) using machine learning techniques. We address this problem with a two-step approach. In the first step, the aim is to identify the patient group with comparably greater severity of speech impairments using Unified Parkinson’s Disease Rating Scale (UPDRS) score as an index of disease progression [29]. We utilize three supervised learning approaches to determine this patient group. We also apply two unsupervised approaches to validate the results obtained with the classification procedure. Then, in the second step of our approach, we exclude the samples of this group of patients with severe speech disorders from the dataset and create a new dataset consisting of the samples of PD patients with mild speech disorders and healthy subjects. We feed this dataset to three different classifiers and present the results in detail. Thus, we aim to analyze the usefulness of vocal features in discriminating the PD patients with early signs of speech disorders and healthy subjects. The highest accuracy of 96.4% and Matthew’s Correlation Coefficient of 0.77 obtained using SVM with third-degree polynomial kernel show that vocal features are effective in discriminating healthy subjects and PD patients with mild speech disorders and can be used for early telediagnosis of the disease.

## Materials and methods

### Dataset description

In the first step of our approach, we use a Parkinson's Disease (PD) telemonitoring dataset consisting of speech recordings of 42 PD patients, which was collected under the supervision of six U.S. medical centers within the context of Tsanas et al.’s study [8] and is available online at UCI machine-learning archive [30]. As described in [8], the data were collected remotely at the patient's home and transmitted over the internet [8]. The PD patients were diagnosed within the previous five years at trial onset if he/she had at least two of the following symptoms: rest tremor, bradykinesia or rigidity, without evidence of other forms of parkinsonism. The patients’ ages ranged from 36 to 85 years (mean age 64.4 ± 9.24) [8]. More detailed description of the data collection process can be found in [8]. The feature set extracted from the voice recordings consists of 16 features which are shown in Table 1 [8, 30, 31]. The statistical parameters of these features are given in Table 2. The feature set includes several measures of fundamental frequency, several measures of variation in amplitude, noise-to-harmonics and harmonics-to-noise ratios, nonlinear dynamical complexity measure, signal fractal scaling exponent, and pitch period entropy. The PD patients were monitored for a six-month period, and remained un-medicated during the duration of the study [8]. The voice recordings of the subjects were obtained at weekly intervals for the six-month duration of the study whereas motor and total UPDRS were assessed only three times by the medical staff: at baseline (onset of trial), and after three and six months. The missing weekly UPDRS estimates corresponding to the weekly voice recordings were obtained using linear interpolation [8]. During the six months data collection period, in each trial, six sustained phonations of the vowel /a/ were recorded summing up to 5875 voice recordings. The motor UPDRS score of the PD patients monitored in this study range from 5 to 40. The control group dataset used in the second step of our analysis is a part of another PD dataset which was collected by Little et al. [23] and is also online available at UCI machine-learning archive [32]. It contains 48 samples belonging to 8 healthy subjects. We refer the reader to [23] for more detailed description of the dataset containing the healthy subjects.

**Table 1:** Definitions of vocal features.

Vocal Feature	Description
Jitter(%)	Average absolute difference between consecutive periods, divided by the average period.
Jitter(Abs)	Average absolute difference between consecutive periods which gives information about the cycle-to-cycle variation of fundamental frequency given in seconds.
Jitter:RAP	Relative Average Perturbation (RAP), which is the average absolute difference between a period and the average of it and its two neighbours, divided by the average period.
Jitter:PPQ5	Five-point Period Perturbation Quotient, computed as the average absolute difference between a period and the average of it and its four closest neighbours, divided by the average period.
Jitter:DDP	Average absolute difference between consecutive differences between consecutive periods, divided by the average period.
Shimmer	Average absolute difference between the amplitudes of consecutive periods, divided by the average amplitude.
Shimmer(dB)	Average absolute base-10 logarithm of the difference between the amplitudes of consecutive periods, multiplied by 20. It gives information about the variability of the peak-to-peak amplitude in decibels.
Shimmer:APQ3	Three-point Amplitude Perturbation Quotient, the average absolute difference between the amplitude of a period and the average of the amplitudes of its neighbours, divided by the average amplitude.
Shimmer:APQ5	Five-point Amplitude Perturbation Quotient, the average absolute difference between the amplitude of a period and the average of the amplitudes of it and its four closest neighbours, divided by the average amplitude.
Shimmer:APQ11	11-point Amplitude Perturbation Quotient, the average absolute difference between the amplitude of a period and the average of the amplitudes of it and its ten closest neighbours, divided by the average amplitude.
Shimmer:DDA	Average absolute difference between consecutive differences between the amplitudes of consecutive periods.
Noise to Harmonics Ratio (NHR)	Amplitude of noise relative to tonal components. It quantifies the noise which occurs due to turbulent airflow, resulting from incomplete vocal fold closure in speech pathologies.
Harmonics to Noise Ratio	Amplitude of tonal relative to noise components. It has the same aim as NHR.
Recurrence period density entropy	Addresses the ability of the vocal folds to sustain stable vocal fold vibrations, quantifying the deviations from exact periodicity
Detrended fluctuation analysis	Quantifies the self-similarity of the noise present in the speech caused by the turbulent air flow
Pitch period entropy	Measures the impaired control of stable pitch during sustained phonations

**Table 2:** Statistical parameters of vocal features.

	Vocal Feature	Minimum	Maximum	Median	Mean	Std. Dev.
**Several measures of variation in fundamental frequency**	Jitter(%)	0.0008	0.1000	0.0049	0.0062	0.0056
	Jitter(Abs)	0	0.0004	0	0.0000	0.0001
	Jitter:RAP	0.0003	0.0575	0.0022	0.0030	0.0031
	Jitter:PPQ5	0.0004	0.0696	0.0025	0.0033	0.0037
	Jitter:DDP	0.0010	0.1726	0.0067	0.0090	0.0094
**Several measures of variation in amplitude**	Shimmer	0.0031	0.2686	0.0275	0.0340	0.0258
	Shimmer(dB)	0.0260	2.1070	0.253	0.3110	0.2303
	Shimmer:APQ3	0.0016	0.1627	0.0137	0.0172	0.0132
	Shimmer:APQ5	0.0019	0.1670	0.0159	0.0201	0.0167
	Shimmer:APQ11	0.0025	0.2755	0.0227	0.0275	0.0200
	Shimmer:DDA	0.0048	0.488	0.0411	0.0515	0.0397
**Two measures of ratio of noise to tonal components in the voice**	Noise to Harmonics Ratio	0.0003	0.7483	0.0184	0.0321	0.0597
	Harmonics to Noise Ratio	1.6590	37.875	21.92	21.6795	4.2911
**A nonlinear dynamical complexity measure**	Recurrence period density entropy	0.1510	0.9661	0.5423	0.5415	0.1010
**Signal fractal scaling exponent**	Detrended fluctuation analysis	0.5140	0.8656	0.6436	0.6532	0.0709
**A nonlinear measure of fundamental frequency variation**	Pitch period entropy	0.0220	0.7317	0.2055	0.2196	0.0915

### Determination of UPDRS threshold

#### Binary classification

In our two-step approach, first we aim to determine the optimal motor UPDRS threshold value that can be discriminated with the lowest possible error rate using vocal features. This threshold value represents the level of disease as determined by UPDRS, after which dysphonia or change in voice quality becomes apparent. For this purpose, we discretize the UPDRS scores of PD patients into two classes, “Below threshold” and “Above threshold”, for various motor UPDRS threshold values. The interval of the UPDRS threshold value that has been evaluated is determined so that each of the classes contains at least 10% of the total number of samples. For each possible optimum threshold value, we apply a binary classification procedure to discriminate the PD patients having UPDRS values below the determined threshold, labeled “negative”, and above the determined possible threshold, labeled “positive [29].

The features are fed to Support Vector Machines (SVM), Extreme Learning Machines (ELM), and *k-near*est neighbors (*k*-NN) classifiers for each of the binary classification problems obtained with various UPDRS threshold values. Although we present the results in terms of accuracy and Matthew’s Correlation Coefficient (MCC) evaluation metrics, since the binary classification problem obtained according to the determined UPDRS threshold value may result in imbalanced datasets in which sample from one class is in higher number than other, we take the MCC metric into account to determine the maximally predictable UPDRS threshold value. The MCC metric is a balanced measure which can be used even if the classes are of very different sizes. It gets a value between –1 and +1. The formulation of MCC metric is given below:

$$MCC=\frac{TP\times TN-FP\times FN}{\sqrt{\left(TP+FP)(TP+FN)(TN+FP)(TN+FN\right)}}$$ (1)

where TP and TN represents the number of correctly classified positive and negative examples, respectively, and FP and FN represents the number of incorrectly classified positive and negative examples, respectively. MCC gets the value of +1 when the classifier makes perfect predictions, –1 when the predictions and actual values totally disagree, and 0 when the classification is no better than a random prediction.

#### Principal component analysis visualization

Principal Component Analysis (PCA) is an unsupervised dimensionality reduction method which projects the samples onto a series of orthogonal axes while preserving as much as possible of the variation present in the dataset [33]. The reduced space consists of the linear combinations of the interrelated variables. We apply PCA to the Parkinson's disease dataset and projected samples onto the PCA subspace with 3 dimensions. We visualize the distributions of the samples for different UPDRS thresholds to see how well the samples above and below these thresholds are discriminated in this reduced space.

#### Clustering analysis

We perform an additional cluster analysis to validate the optimal UPDRS threshold results obtained with the binary classification approach. For this purpose, we use spectral clustering method which uses eigen-values of similarity matrix of the data to reduce the dimension as a pre-processing step before clustering [34]. In this method, each sample is represented with several components in the corresponding eigen-vectors of the similarity matrix and this reduced space is fed to another clustering algorithm. In this study, we construct the *k-*nearest neighbors similarity graph of the samples and feed this similarity matrix to *k*-means clustering method. We divide the samples into two clusters and analyze the UPDRS scores of the patients in each cluster for various UPDRS threshold values.

### Feature ranking based on determined optimal UPDRS threshold

We aim to quantify the relevance of each vocal feature with the discretized UPDRS score using Mutual Information (MI) [35]. Thus, the vocal features which significantly change with respect to the level of motor systems disorders as determined by UPDRS will be identified. The MI approach used in this study is a filter method which aims to rank the features according to their relevance with the target variable without involving any classifier/regressor for evaluation [36–38].

The mutual information is a measure of mutual dependence of the two variables which can also capture non-linear relations. It is based on Shannon’s entropy [35] which is a measure of the uncertainty of a random variable *X* and thus, it quantifies how difficult it is to predict that variable. The definition of Shannon's entropy can be written as an expectation:

$$H(X)=-E[\text{log}P(X)]|=-\sum_{x}\left[p(x)\text{log}(p(x))\right]$$ (2)

where *p*(*x*) = *P*(*X* = *x*) is the probability distribution function of *X*. Hence the Shannon's entropy represents the uncertainty removed after the actual outcome of *X* is revealed. MI is a measure of mutual dependence of the two variables based on the entropy:

I(X;Y)=H(X)+H(Y)−H(X,Y) (3)

MI is also the KL divergence of the product *P(X)P(Y)* of the two marginal probability distributions from the joint probability distribution, *P(X*,*Y)*.

$$I(X;Y)=D_{KL}(P(X,Y)||P(X)\cdot P(Y))=\sum_{x}\sum_{y}\left[p(x,y)\text{log}(\frac{p(x,y)}{p(x)\cdot p(y)})\right]$$ (4)

where *p*(*x*,*y*) = *P*(*X* = *x*, *Y* = *y*). As described in Materials and methods, the features of the PD dataset are continuous. Therefore, to compute the MI scores between the features and the discretized UPDRS, we discretize the features to 9 discrete levels [36, 38]. For discretization, for each feature, we use its mean μ and its standard deviation σ as in [37]. The feature values between μ–σ/2 and μ+σ/2 are converted to 0. The 4 intervals of size σ to the right of μ+σ/2 are converted to discrete levels from 1 to 4 and the 4 intervals of size σ to the left of μ−σ/2 are mapped to discrete levels from −1 to −4. Very large positive or negative feature values are truncated and discretized to ±4 appropriately.

### Discrimination between healthy subjects and patients with UPDRS below threshold

After determining the optimal UPDRS threshold value that can be discriminated using the vocal features, we exclude the samples of the patients whose motor UPDRS score is above this threshold and created a new dataset consisting of the samples of PD patients whose UPDRS score is below this threshold and 8 healthy subjects. The aim of this analysis is to evaluate the effectiveness of speech features in discriminating the early stage PD patients and healthy subjects. We use SVM, ELM, and *k*-NN classifiers and present the accuracy and MCC of each classifier for different parameter values and kernel types.

## Experimental results

### Determination of optimal UPDRS threshold

#### Binary classification problem

**Fig 1:**
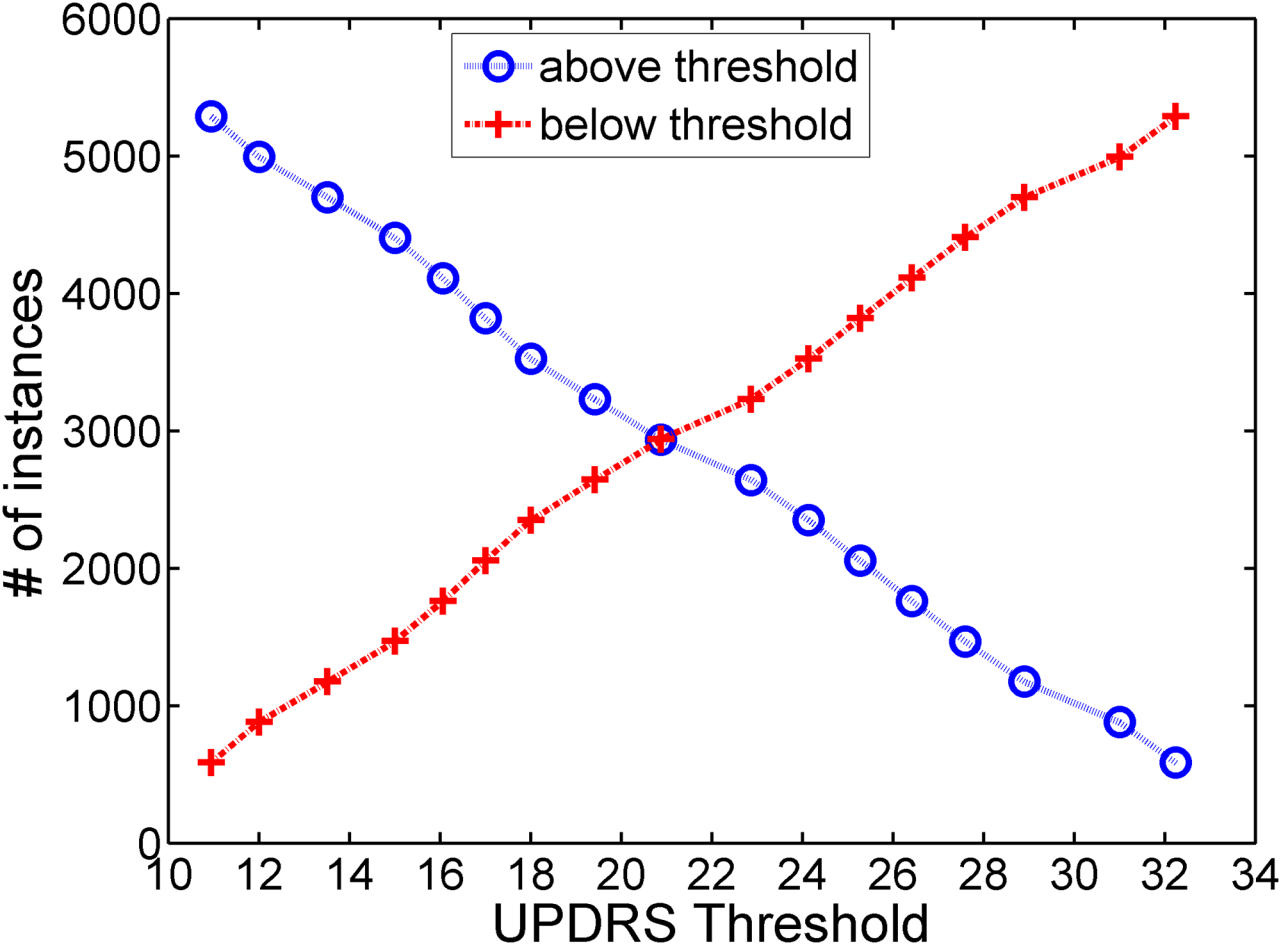
Number of positive (above threshold) and negative instances (below threshold) with respect to determined UPDRS threshold.

We first normalize the features of the PD dataset so that each has a zero mean and unit variance. Then, the features are fed into SVM, ELM and *k*-NN classifiers for various motor UPDRS threshold values. We use 70% of the samples for training and the rest for validation. For *k*-NN classifier, we use Euclidean, city-block, and correlation as distance metrics, varying the number of nearest neighbors (*k*) from 1 to 9. We present the *k*-NN results only with city-block distance since it performed better than or comparable to the other distance metrics. For SVM classifier, we use LIBSVM implementation [39] with linear, polynomial, and Radial Basis Function (RBF) kernels, varying the cost (C) parameter from 0.1 to 10 increasing by 0.2, polynomial degree from 1 to 5, and kernel width (g) from 0.01 to 1 increasing by 0.02. As it is seen in Fig 1, depending on the value of the UPDRS threshold value, the PD dataset becomes highly imbalanced, and SVM classifier tends to label the samples as majority class to minimize the error on the training set. We use the "class-weight" parameter of LIBSVM, *w*, to address the class imbalance problem. The class weight parameter of SVM is used to increase the cost of errors made on the samples of minority class during training. In the implementation of ELM classifier, we vary the number of hidden neurons from 50 to 200 with sigmoid, sine, RBF, and triangular basis activation functions.

**Fig 2:**
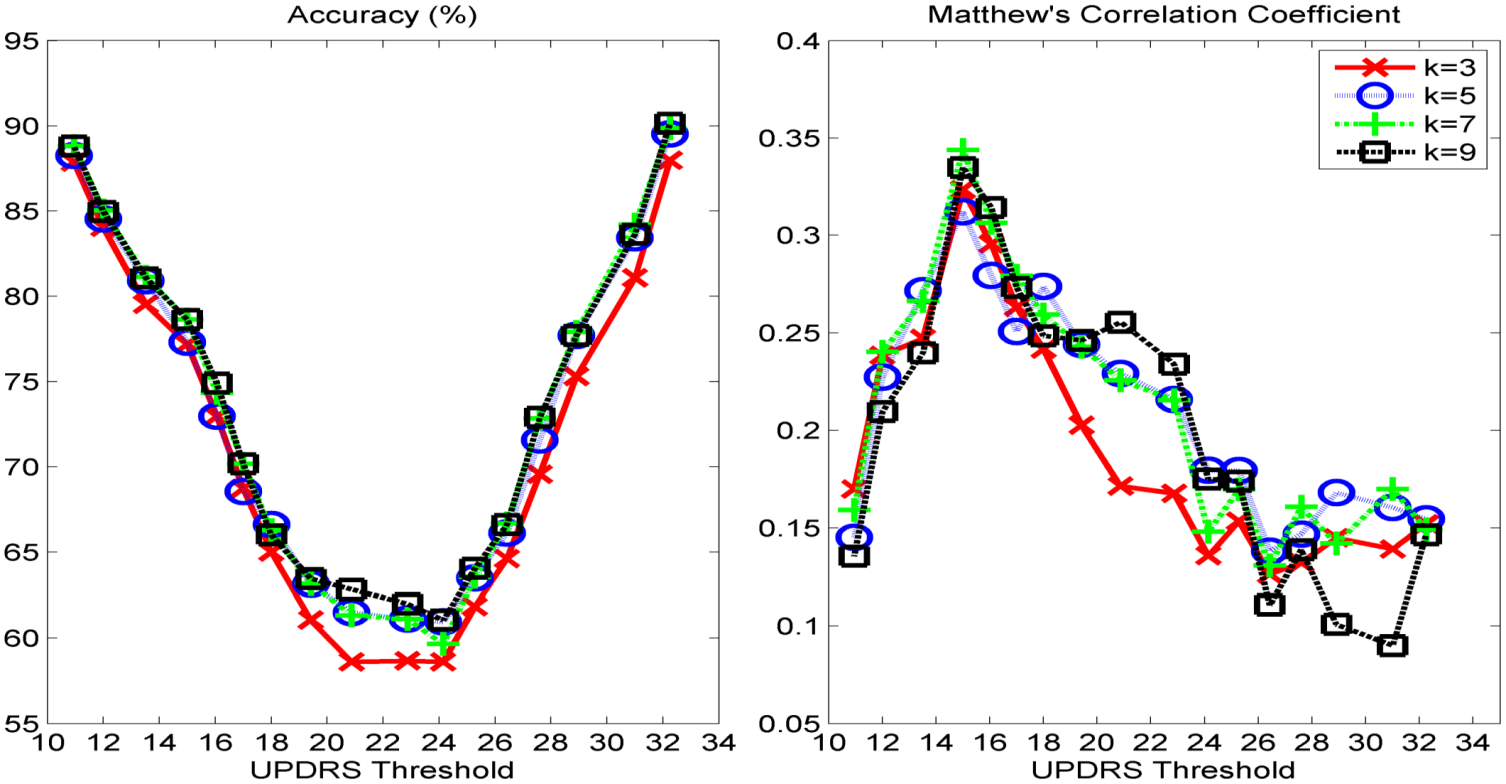
(left) Test set classification accuracies and (right) Matthew's correlation coefficients obtained with k-NN classifier under various UPDRS threshold values.

**Fig 3:**
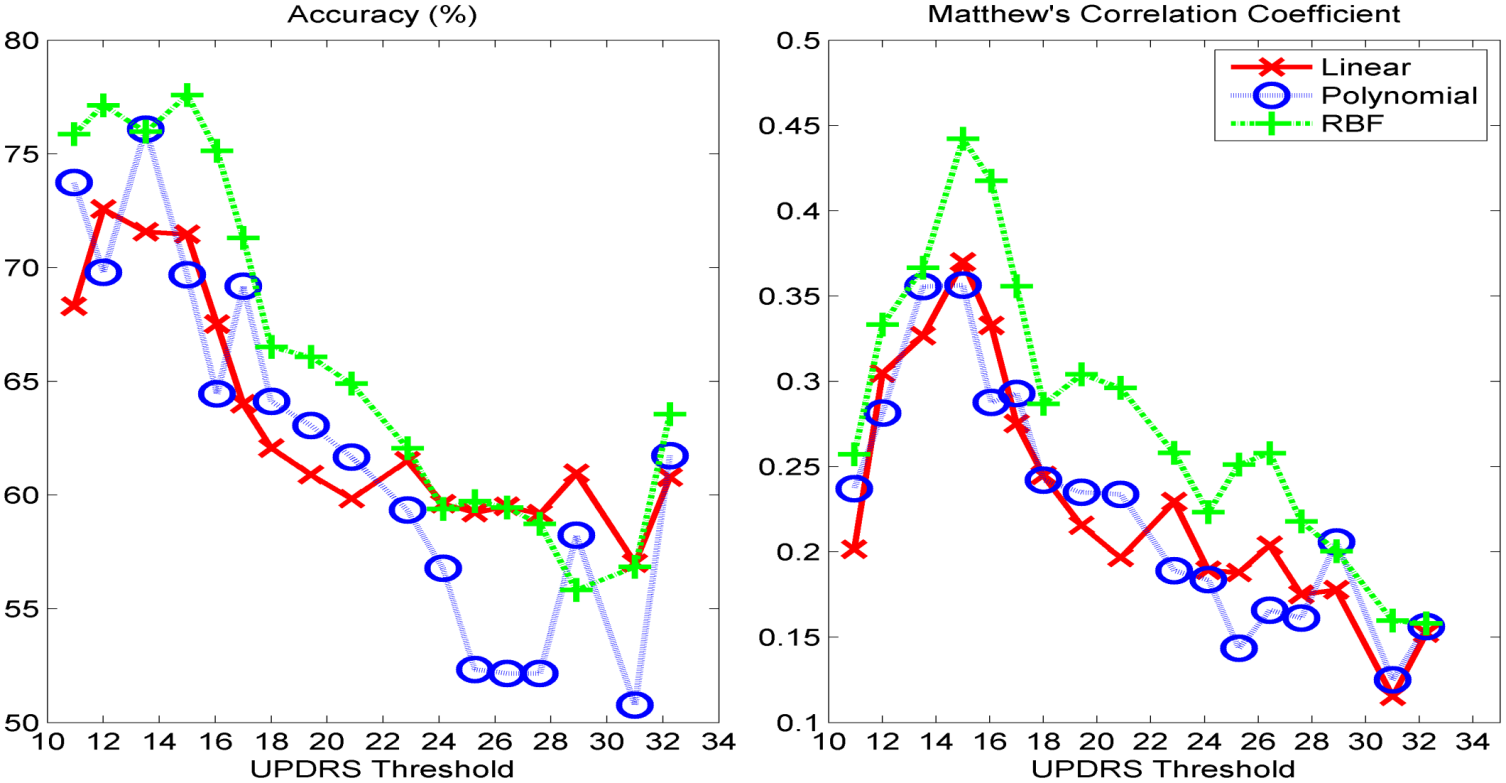
(left) Test set classification accuracies and (right) Matthew's correlation coefficients obtained with SVM classifier under various UPDRS threshold value.

**Fig 4:**
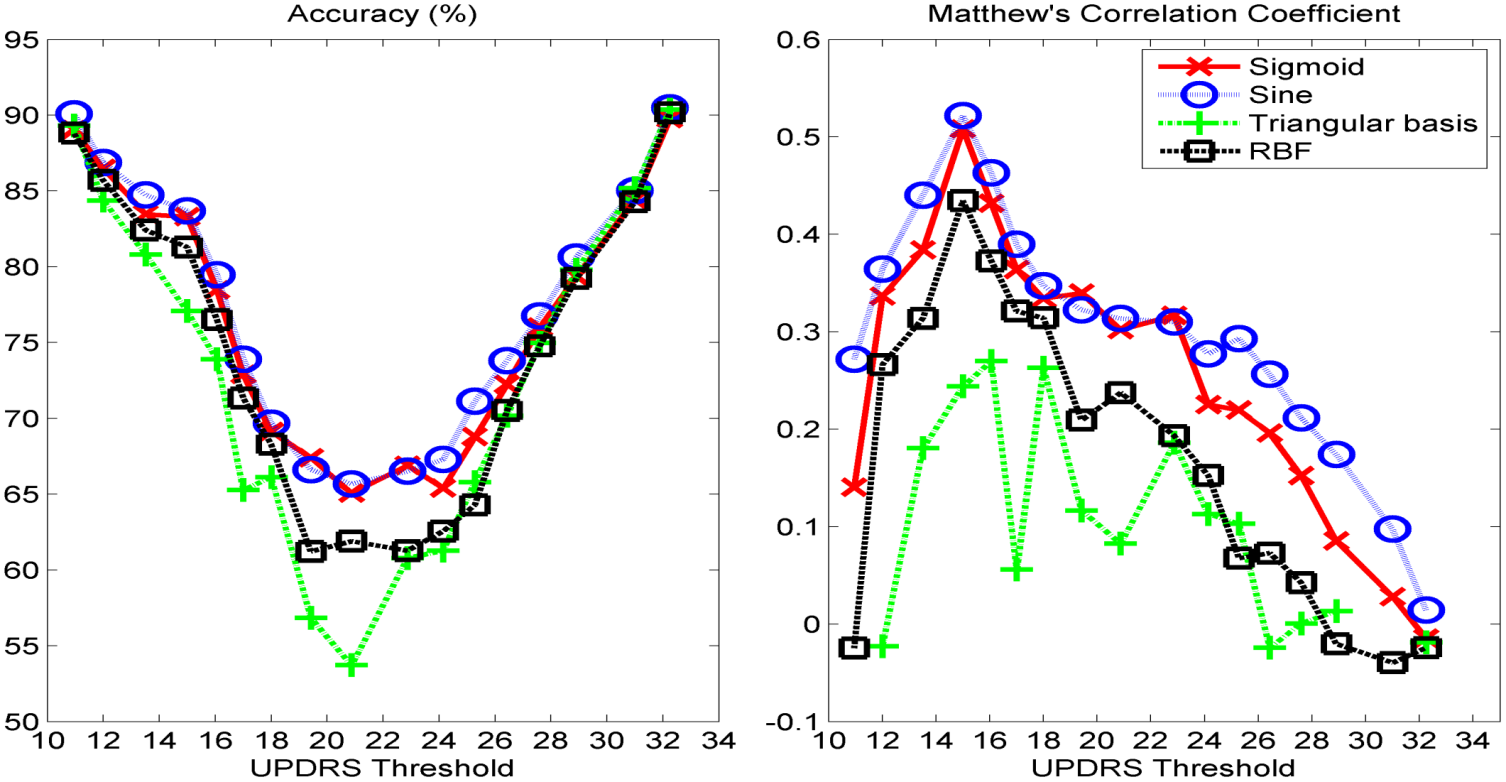
(left) Test set classification accuracies and (right) Matthew's correlation coefficients obtained with ELM classifier under various UPDRS threshold values.

We present the classification performance of *k*-NN using cityblock distance, ELM with sigmoid, sine, triangular basis, and RBF activation functions, and SVM with linear and Radial Basis Functions (RBF) kernels for different UPDRS thresholds. In Figs 2–4, the accuracies and MCC values obtained with the optimal parameter values of the classifiers are shown. As it is seen in Figs 2 and 4, the accuracies of both *k*-NN and ELM classifiers decreases as UPDRS threshold increases up to around 23 and then begins to increase. The highest overall accuracies (~90%) with both of these classifiers are obtained when UPDRS threshold is set to 11 and 32. However, as it is seen in Fig 1, the binary classification problems obtained by setting UPDRS threshold to these values are highly imbalanced (e.g. in test set number of positive examples is 1868 whereas number of negative examples is 207 when UPDRS threshold is set to 11), and using accuracy on such imbalanced datasets may lead to false inferences regarding the success of classifiers [40, 41]. For example, with the class distribution corresponding to UPDRS threshold of 11, a simple strategy of labeling all the test set examples as positive class gives an accuracy of 90.02%. Indeed, it is seen that the accuracy plots of *k*-NN and ELM classifiers are not in compatible with their MCC plots. The MCCs of *k*-NN and ELM increase as UPDRS threshold increases up to around 15 and thereafter shows a decreasing trend. These results show that *k*-NN and ELM tend to label most of the examples as the majority class for the UPDRS thresholds resulted in imbalanced dataset. On the other hand, as seen in Fig 3, the accuracy and MCC performances of SVM-linear and SVM-RBF classifiers change similarly with respect to the UPDRS threshold value. These results show that SVM performs more consistently than *k*-NN and ELM on imbalanced datasets when the costs of errors made on the training samples of majority and minority class are tuned well using its class weight parameter.

**Fig 5:**
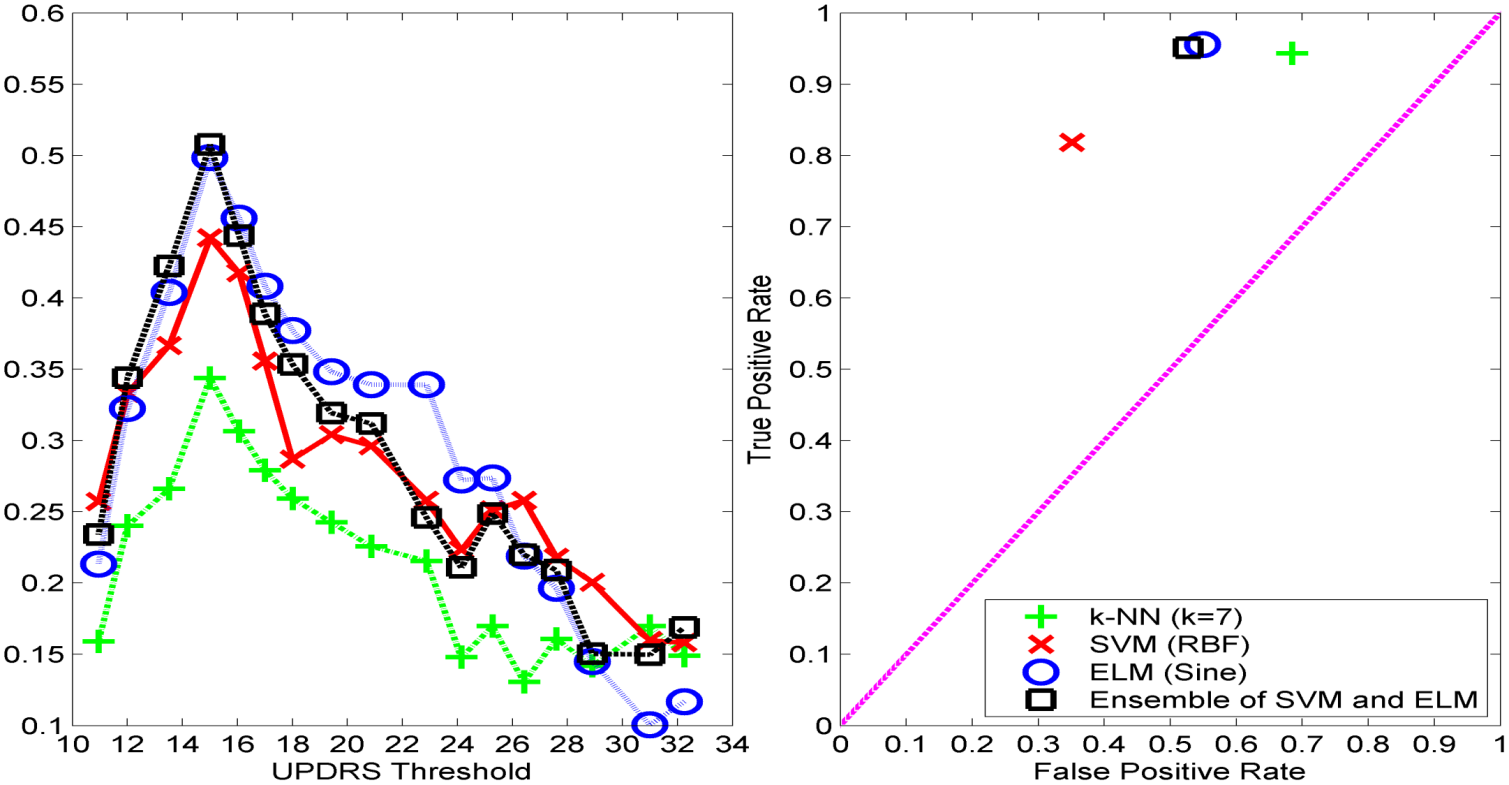
A summary of results obtained with the best settings of classifiers (left) Matthew's correlation coefficients of the classifiers obtained with their best settings (right) ROC space of the classifiers obtained when UPDRS threshold is set to 15.

As seen in Fig 2, *k*-NN performs the highest MCC with *k* = 7 (city-block distance) when UPDRS threshold is set to 15. Similarly, SVM and ELM show their best performance with RBF kernel and sine activation function, respectively, when UPDRS threshold is set to 15. In Fig 5 (left), MCC obtained with the optimal parameter set of each classifier with respect to UPDRS threshold is shown. These results show that UPDRS value of 15 is the optimal threshold value that can be used to monitor the progression of the disease as a classification problem. Fig 5 (left) shows the MCC of all classifiers obtained with their best settings with respect to UPDRS threshold. It is seen that ELM with sine activation function gives the highest MCC (0.5219). The corresponding test set accuracy of ELM is 83.70%. The highest MCC obtained with SVM-RBF (0.4421) is higher than that of *k*-NN (0.3439). The corresponding accuracies of SVM-RBF and *k*-NN are 77.59% and 78.64%, respectively. In Fig 5, the performance of ensemble classifier obtained by combining the predictions of SVM-RBF and ELM-Sine using hard-voting combination strategy [42] is also shown. It is seen that while the performance of SVM-RBF improves when combined with ELM-Sine, ELM-Sine individually performs better than or comparably to the ensemble of SVM-RBF and ELM-Sine. On the other hand, the ROC space of the classifiers shown in Fig 5 (right) obtained when UPDRS threshold is set to 15 shows that although MCC of ELM-Sine is higher than that of SVM-RBF, SVM-RBF is more balanced in correctly classifying the positive and negative instances.

#### Principal component analysis visualization

**Fig 6:**
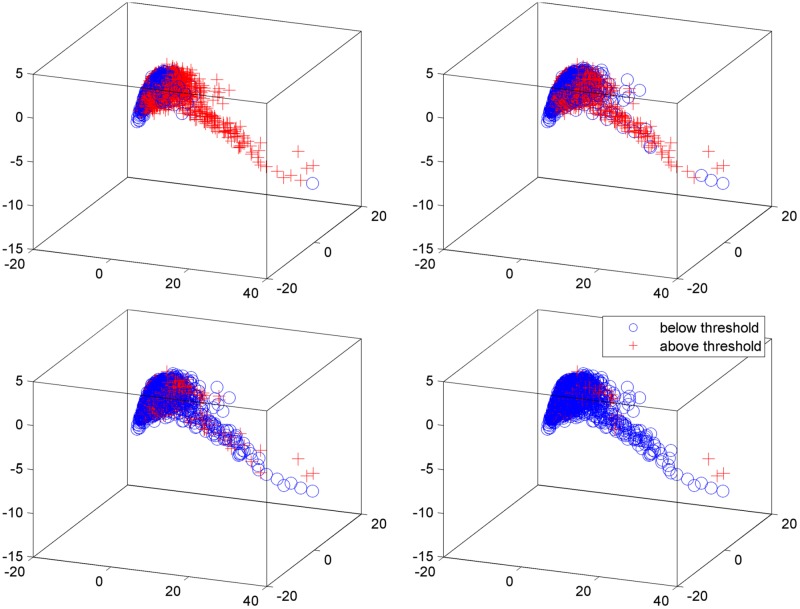
Scatter of PD data on the first three principal components with UPDRS threshold value of (top) (left) 15 (right) 20 (bottom) (left) 25 (right) 30.

We apply principal component analysis (PCA) and reduce the dimensionality of the dataset to visually validate the results obtained with the designed binary classification problem. Fig 6 shows the scatter of PD data on the first three principal components with various UPDRS threshold values. As seen in Fig 6, the projections of the samples belonging to the patients with lower UPDRS score than 15 form a cluster in a region of the PCA subspace. Although they mostly overlap with the other group with UPDRS score higher than 15, it is seen that there is a distinct set of samples with UPDRS score above 15 that does not overlap with the other group. However, with increasing UPDRS threshold, it is seen that number of overlapping samples from the two groups increases. Fig 6 also shows that when UPDRS threshold is set to 25, none of the groups form a separate cluster in any specific region of the PCA subspace. These results validate the findings explored with the binary classification problem.

#### Clustering analysis results

We apply spectral clustering with the settings described in Materials and methods to divide the dataset into two groups in an unsupervised manner. The aim is to compare the UPDRS values of the patients that fall into these two groups. For this purpose, we divide the samples into two clusters by constructing the *k-*nearest neighbors similarity graph of the samples and feeding this similarity matrix to *k*-means clustering method. Then, we analyze the UPDRS scores of the patients in each cluster for various UPDRS threshold values.

**Fig 7:**
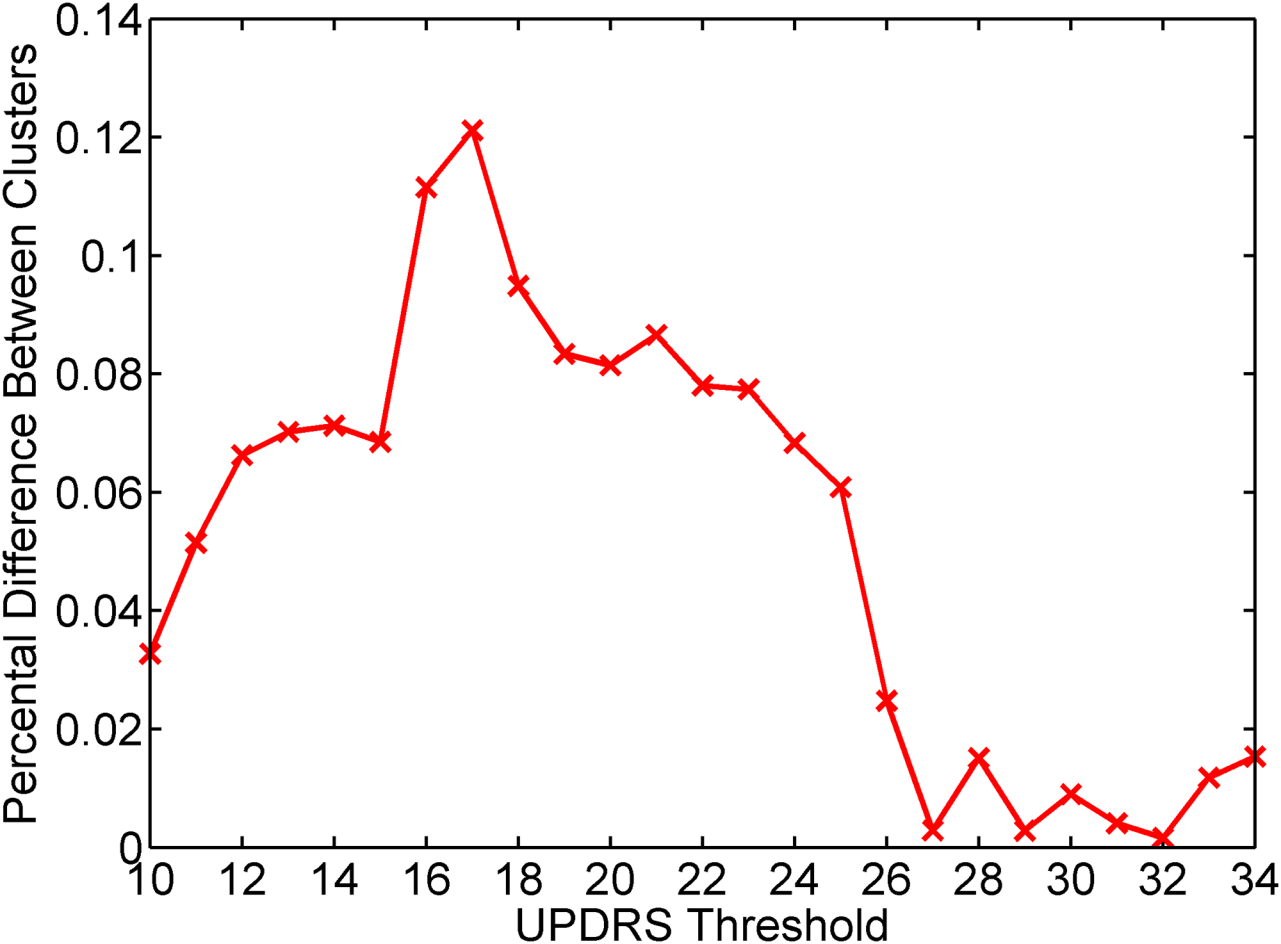
Absolute difference between the ratio of the number of patients whose UPDRS is below the corresponding threshold to the number of all patients in cluster 1 and cluster 2.

The clustering algorithm groups 4307 of the speech recordings in cluster 1 and the rest (1586 recordings) in cluster 2. After obtaining the cluster indexes, for each cluster we compute the ratio of the number of patients whose UPDRS score is below the corresponding threshold to the number of all patients in that cluster. Fig 7 shows the absolute difference between the ratios of cluster 1 and cluster 2 for various UPDRS thresholds. It is seen that the highest difference is obtained for UPDRS threshold value of 17 which is very close to the UPDRS threshold of 15 determined with binary classification problem and PCA analysis.

### Feature ranking based on optimal UPDRS threshold

After determining the optimal UPDRS threshold value and converting the UPDRS prediction problem to a binary classification problem using the optimal threshold, we quantify the relevance of each vocal feature with the discretized UPDRS score to reveal which vocal features are related with the severity of UPDRS score. For this purpose, we calculate the Mutual Information (MI) between each of the vocal feature and the discretized UPDRS value.

The ranking of vocal features based on their MI score with the target variable is shown in Table 3. As seen, Detrended Fluctuation Analysis (DFA) which represents the signal fractal scaling exponent is the most effective feature in discriminating the patients with severe motor systems disorders from those who have relatively less severe motor system disorders. DFA is followed by Pitch Period Entropy (PPE), Recurrence Period Density Entropy (RPDE), and Harmonics to Noise Ratio (HNR), respectively. We should note PPE is found as one of the most relevant vocal features in discriminating the healthy subjects from PD patients in [21] on a PD dataset consisting of speech recordings of 23 patients and 8 healthy subjects. However, the findings in [21] show that Jitter:DDP, Shimmer, Shimmer(dB), and Shimmer:APQ5 are more important in healthy subject/PD patient discrimination problem than DFA, RPDE, and HNR.

**Table 3:** Ranking of the vocal features based on their mutual information with UPDRS level discretized according to the determined optimal threshold that can be discriminated by machine learning methods.

Ranking	Dysphonia Measurement	MI Score
1	DFA	0.0413
2	PPE	0.0302
3	RPDE	0.0287
4	HNR	0.0277
5	NHR	0.0202
6	Jitter:PPQ5	0.0189
7	Jitter(%)	0.0189
8	Jitter(Abs)	0.0163
9	Shimmer:APQ11	0.0163
10	Jitter:DDP	0.0155
11	Jitter:RAP	0.0154
12	Shimmer(dB)	0.0120
13	Shimmer	0.0112
14	Shimmer:APQ3	0.0084
15	Shimmer:DDA	0.0084
16	Shimmer:APQ5	0.0081

### Discrimination between healthy subjects and patients with UPDRS below threshold

The supervised learning problem (binary classification) has been designed to determine the UPDRS value after which speech disorders begin to emerge. The unsupervised approaches (PCA analysis and spectral clustering) results have validated the determined UPDRS threshold value. In the second step, we exclude the samples of the patients whose motor UPDRS score is above this threshold and include the samples of healthy subjects. Thus, we create a new dataset consisting of all speech recordings of the healthy subjects and 1607 speech recordings of the PD patients. Then, we apply k-NN, SVM and ELM classifiers with various settings using 70% of dataset for training and the rest for validation to evaluate the effectiveness of vocal features in discriminating the early stage PD patients and healthy subjects. For statistical significance, this procedure is repeated 100 times with random training/test partitions. We present the average and standard deviation of the accuracies of these runs for each classifier in Table 4. The best results obtained with k-NN, SVM and ELM are given in Table 5 along with the p-values of paired t-test for each pair of classifiers. As it is seen, the accuracy (96.43%) and MCC (0.77) of SVM with third-degree polynomial are significantly higher than that of the other methods. It is also seen that correlation distance based k-NN significantly outperformed ELM in terms of MCC which shows that it has produced more balanced success rates on positive and negative instances. On the other hand, the lowest accuracy is obtained with liner kernel SVM which is the only linear classifier used in this study. We should also note that healthy subjects and patients with UPDRS score lower than 15 are better discriminated with vocal features than the patients with below and above determined UPDRS score are.

**Table 4:** Accuracies and MCC values obtained with various settings of *k*-NN, SVM, and ELM on the dataset consisting of the samples of PD patients whose UPDRS is below this threshold and 8 healthy subjects.

k-NN (k = 3)			SVM			ELM		
Distance	Accu. (%)	MCC	Kernel	Accu (%)	MCC	Kernel	Accu. (%)	MCC
**Cosine**	94.69±0.01	0.63±0.10	**Linear**	89.38±0.02	0.58±0.06	**Sigmoidal**	90.07±0.02	0.51±0.09
**Correlation**	94.99±0.01	0.65±0.11	**Polynomial**	**96.43±0.01**	**0.77±0.09**	**Sine**	91.93±0.02	0.56±0.09
**Cityblock**	93.30±0.01	0.40±0.14	**RBF**	91.86±0.02	0.63±0.07	**RBF**	91.93±0.02	0.41±0.16

**Table 5:** Best results obtained with k-NN, SVM and ELM with statistical significance tests.

	k-NN (1)	SVM (2)	ELM (3)	Statistical Significance 1–2	Statistical Significance 1–3	Statistical Significance 2–3
**Accu. (%)**	94.99	96.43	91.93	**	**	*
**MCC**	0.65	0.77	0.56	*	*	*

Paired t-test:

** p < 0.01;

* p < 0.05

### Conclusions

Considering that PD mostly targets the elderly people whose physical visits to the clinic are inconvenient and costly, there is an increasing motivation to develop PD telemonitoring and telediagnosis systems which are self-administrated and do not require the patient’s visit to the clinic. Since the vocal impairments are one of the most commonly seen PD signs in the early stages of the disease, the PD telediagnosis and telemonitoring systems based on speech tests result in reliable diagnosis and motor UPDRS tracking systems. However, the patient data used in the existing telediagnosis systems include speech recordings of not only early PD patients with mild speech impairments but also PD patients with moderate and severe speech impairments who already suffer from some other symptoms and presumably have been diagnosed before. In this paper, we aim to assess the effectiveness of vocal features for early telediagnosis of PD in a more realistic scenario. First, as a preprocessing step, we first determine the group of patients with relatively greater speech impairments using Unified Parkinson’s Disease Rating Scale (UPDRS) as an index of disease progression. For this purpose, we discretize the UPDRS scores of PD patients into two classes, “Below threshold” and “Above threshold”, for various motor UPDRS threshold values, and for each case apply a binary classification procedure to discriminate the PD patients having UPDRS values below the determined threshold, labeled “negative”, and above the determined possible threshold, labeled “positive”. The UPDRS value resulting the highest classification performance is chosen as the UPDRS threshold value, after which the speech disorders are more significantly seen in the patients. We validate the determined threshold value with two unsupervised approaches: principal component analysis (PCA) and spectral clustering. The experimental results show that speech disorders are more significantly seen in the PD patients whose UPDRS exceeds 15. Considering that the motor UPDRS ranges from 0 to 108, relatively low UPDRS threshold of 15 shows that vocal impairments can be used as early indicators of the disease. The highest Matthew’s correlation coefficient (MCC) is obtained using support vector machines (SVM) with radial basis functions (RBF) kernel, which also gives higher MCC values than k-nearest neighbors (k-NN) and SVM with linear kernel for all UPDRS threshold values. Besides, we should also note that SVM performs more consistently than k-NN and extreme learning machines classifiers on PD dataset with imbalanced class distribution when the costs of errors made on the training samples of majority and minority class are tuned well using its class weight parameter. The mutual information based filter feature ranking analysis show that nonlinear feature extraction methods named as detrended fluctuation analysis and pitch period entropy are the most effective speech features in discriminating the patients with severe motor systems disorders from those whose motor system disorders are relatively less severe. The visual inspection presented using PCA also shows that simple lines or hyperplanes cannot discriminate the two groups from each other. These results strongly indicate the nonlinearity behavior of the problem and, therefore, it needs to be solved by a nonlinear model.

In the second step, to address the main goal of this paper, we exclude the speech recordings of the PD patients having higher UPDRS score than the determined threshold in the first step and create a new dataset consisting of the samples of PD patients whose UPDRS is below the determined threshold value and healthy subjects. Thus, we assess the PD telediagnosis ability of vocal features in a more realistic scenario for clinical use. We feed this dataset into three classifiers and present the detailed results. For best generalization, the complexity of the classifier should match the complexity of the function underlying the data [43]. More complex models than the underlying function can lead to overfitting in which the model identifies random noise in the data, rather than a true signal of clinical use (Sachs, 2015), whereas models that are less complex than the function can lead to underfitting. Therefore, to evaluate the generalization ability of the classifiers, the hyperparameters such as the number of nearest neighbors of k-NN or degree and cost of polynomial kernel-SVM should be optimized on a separate set that has not been used during training. For this purpose, we train three classifiers with various settings using 70% of dataset for training and use the rest for validation to evaluate the effectiveness of vocal features in discriminating the early stage PD patients and healthy subjects. For statistical significance, this procedure is repeated 100 times with random training/test partitions and paired t-test is applied to test the statistical significance of the results. The highest accuracy of 96.4% and Matthew’s Correlation Coefficient of 0.77 is obtained using SVM with third-degree polynomial kernel. Lower and higher degree values than this optimal value cause less accuracies due to the model’s simplicity and overfitting problems, respectively. These results show that speech features are effective in discriminating PD patients with mild speech impairment from healthy subjects and can be used as a decision support system for early telediagnosis of the disease. The success of SVM with nonlinear kernels in PD classification problem is not surprising as it has already been shown in the literature [21, 23, 44]. Considering that many of the speech signals are noisy [23], we can conclude that, as shown on related audio processing problems from different domains [45–48], SVM with a non-liner kernel produces more generalizable models which are robust to noise and outliers compared to many classification algorithms such as those used in this study. On the other hand, we should note that the lowest accuracy is obtained with liner kernel SVM which is the only linear classifier used in this study. This is mainly because non-linear relationships between the vocal features in UPDRS prediction are overlooked by a linear method.

We should note that using motor UPDRS score as the index of disease progression instead of more specific UPDRS subscores representing the severity of speech and other disorders is a limitation of this study. As a future research direction, a dataset containing UPDRS subscores may be collected and used as the index of disease progression to better identify the patient group having mild motor system disorders.

## References

[1] Braak H., & Braak E. (2000). Pathoanatomy of Parkinson’s disease. Journal of neurology, 247, II3–II10. doi: 10.1007/PL00007758 10991663 10.1007/PL00007758

[2] Pringsheim T, Jette N, Frolkis A, Steeves TD. The prevalence of Parkinson's disease: A systematic review and meta‐analysis. Movement disorders. 2014 11 1;29(13):1583–90. doi: 10.1002/mds.25945 24976103 10.1002/mds.25945

[3] Twelves D, Perkins KS, Counsell C. Systematic review of incidence studies of Parkinson's disease. Movement disorders. 2003 1 1;18(1):19–31. doi: 10.1002/mds.10305 12518297 10.1002/mds.10305

[4] von Campenhausen S, Bornschein B, Wick R, Bötzel K, Sampaio C, Poewe W, et al Prevalence and incidence of Parkinson's disease in Europe. European Neuropsychopharmacology. 2005 8 31;15(4):473–90. doi: 10.1016/j.euroneuro.2005.04.007 15963700 10.1016/j.euroneuro.2005.04.007

[5] Pahwa R, Lyons KE. Early diagnosis of Parkinson’s disease: recommendations from diagnostic clinical guidelines. Am J Manag Care. 2010 3 1;16(4):94–9. 20297872

[6] Huse DM, Schulman K, Orsini L, Castelli‐Haley J, Kennedy S, Lenhart G. Burden of illness in Parkinson's disease. Movement disorders. 2005 11 1;20(11):1449–54. doi: 10.1002/mds.20609 16007641 10.1002/mds.20609

[7] Schenkman M, Zhu CW, Cutson TM, Whetten-Goldstein K. Longitudinal evaluation of economic and physical impact of Parkinson's disease. Parkinsonism & related disorders. 2001 9 30;8(1):41–50. 11472879 10.1016/s1353-8020(00)00079-1

[8] Tsanas A, Little MA, McSharry PE, Ramig LO. Accurate telemonitoring of Parkinson's disease progression by noninvasive speech tests. IEEE transactions on Biomedical Engineering. 2010 4;57(4):884–93. doi: 10.1109/TBME.2009.2036000 19932995 10.1109/TBME.2009.2036000

[9] Sakar BE, Isenkul ME, Sakar CO, Sertbas A, Gurgen F, Delil S, Apaydin H, Kursun O. Collection and analysis of a Parkinson speech dataset with multiple types of sound recordings. IEEE Journal of Biomedical and Health Informatics. 2013 7;17(4):828–34. doi: 10.1109/JBHI.2013.2245674 25055311 10.1109/JBHI.2013.2245674

[10] Peacock JH. Department of Veteran Affairs and American Parkinson Disease Association, INC Spring 2016 Parkinson Press Newsletter.

[11] Ramaker C, Marinus J, Stiggelbout AM, van Hilten BJ. Systematic evaluation of rating scales for impairment and disability in Parkinson's disease. Movement Disorders. 2002 9 1;17(5):867–76. doi: 10.1002/mds.10248 12360535 10.1002/mds.10248

[12] Siderowf A, McDermott M, Kieburtz K, Blindauer K, Plumb S, Shoulson I. Test–retest reliability of the unified Parkinson's disease rating scale in patients with early Parkinson's disease: results from a multicenter clinical trial. Movement disorders. 2002 7 1;17(4):758–63. doi: 10.1002/mds.10011 12210871 10.1002/mds.10011

[13] Giladi N, Shabtai H, Simon ES, Biran S, Tal J, Korczyn AD. Construction of freezing of gait questionnaire for patients with Parkinsonism. Parkinsonism & related disorders. 2000 7 31;6(3):165–70. 10817956 10.1016/s1353-8020(99)00062-0

[14] Bayestehtashk A, Asgari M, Shafran I, McNames J. Fully automated assessment of the severity of Parkinson's disease from speech. Computer speech & language. 2015 1 31;29(1):172–85. 25382935 10.1016/j.csl.2013.12.001 PMC4222054

[15] Tsanas A, Little MA, McSharry PE, Ramig LO. Nonlinear speech analysis algorithms mapped to a standard metric achieve clinically useful quantification of average Parkinson's disease symptom severity. Journal of the Royal Society Interface. 2011 6 6;8(59):842–55. 10.1098/rsif.2010.0456 PMC3104343 21084338

[16] Movement Disorder Society Task Force on Rating Scales for Parkinson's Disease. The Unified Parkinson's Disease Rating Scale (UPDRS): status and recommendations. Movement disorders: official journal of the Movement Disorder Society. 2003 7;18(7):738. 12815652 10.1002/mds.10473

[17] Martínez‐Martín P, Gil‐Nagel A, Gracia LM, Gómez JB, Martínez‐Sarriés J, Bermejo F. Unified Parkinson's disease rating scale characteristics and structure. Movement disorders. 1994 1 1;9(1):76–83. doi: 10.1002/mds.870090112 8139608 10.1002/mds.870090112

[18] Harel B, Cannizzaro M, Snyder PJ. Variability in fundamental frequency during speech in prodromal and incipient Parkinson's disease: A longitudinal case study. Brain and cognition. 2004 10 31;56(1):24–9. doi: 10.1016/j.bandc.2004.05.002 15380872 10.1016/j.bandc.2004.05.002

[19] Bennett DA, Shannon KM, Beckett LA, Goetz CG, Wilson RS. Metric properties of nurses' ratings of parkinsonian signs with a modified Unified Parkinson's Disease Rating Scale. Neurology. 1997 12 1;49(6):1580–7. 9409350 10.1212/wnl.49.6.1580

[20] Goetz CG, Stebbins GT, Wolff D, DeLeeuw W, Bronte‐Stewart H, Elble R, Hallett M, Nutt J, Ramig L, Sanger T, Wu AD. Testing objective measures of motor impairment in early Parkinson's disease: Feasibility study of an at‐home testing device. Movement Disorders. 2009 3 15;24(4):551–6. doi: 10.1002/mds.22379 19086085 10.1002/mds.22379 PMC4161502

[21] Sakar CO, Kursun O. Telediagnosis of Parkinson’s disease using measurements of dysphonia. Journal of medical systems. 2010 8 1;34(4):591–9. doi: 10.1007/s10916-009-9272-y 20703913 10.1007/s10916-009-9272-y

[22] Gürüler H. A novel diagnosis system for Parkinson’s disease using complex-valued artificial neural network with k-means clustering feature weighting method. Neural Computing and Applications. 2015:1–10.

[23] Little MA, McSharry PE, Hunter EJ, Spielman J, Ramig LO. Suitability of dysphonia measurements for telemonitoring of Parkinson's disease. IEEE transactions on biomedical engineering. 2009 4;56(4):1015–22. doi: 10.1109/TBME.2008.2005954 21399744 10.1109/TBME.2008.2005954 PMC3051371

[24] Peker M, Sen B, Delen D. Computer-aided diagnosis of Parkinson’s disease using complex-valued neural networks and mRMR feature selection algorithm. Journal of healthcare engineering. 2015;6(3):281–302. 26753436 10.1260/2040-2295.6.3.281

[25] Peker M. A decision support system to improve medical diagnosis using a combination of k-medoids clustering based attribute weighting and SVM. Journal of medical systems. 2016 5 1;40(5):1–6. 27000777 10.1007/s10916-016-0477-6

[26] Sakar BE, Kursun O. Telemonitoring of changes of unified Parkinson’s disease rating scale using severity of voice symptoms." In Proc. 2nd International Conference on E-Health and TeleMedicine, Istanbul, 2014, pp. 114–119.

[27] Khan T, Westin J, Dougherty M. Classification of speech intelligibility in Parkinson's disease. Biocybernetics and Biomedical Engineering. 2014 12 31;34(1):35–45.

[28] Harrison MB, Wylie SA, Frysinger RC, Patrie JT, Huss DS, Currie LJ, Wooten GF. UPDRS activity of daily living score as a marker of Parkinson's disease progression. Movement disorders. 2009 1 30;24(2):224–30. doi: 10.1002/mds.22335 18951537 10.1002/mds.22335 PMC3103833

[29] Sakar BE, Sakar CO, Serbes G, Kursun O. Determination of the optimal threshold value that can be discriminated by dysphonia measurements for unified Parkinson's Disease rating scale. In Bioinformatics and Bioengineering (BIBE), 2015 IEEE 15th International Conference on 2015 Nov 2 (pp. 1–4). IEEE.

[30] Rusz J, Cmejla R, Ruzickova H, Ruzicka E. Quantitative acoustic measurements for characterization of speech and voice disorders in early untreated Parkinson’s disease. The journal of the Acoustical Society of America. 2011 1;129(1):350–67.J. Acoust. Soc. Am., 129 (1) (2011), pp. 350–367 doi: 10.1121/1.3514381 21303016 10.1121/1.3514381

[31] Farrús M. Jitter and shimmer measurements for speaker recognition. In 8th Annual Conference of the International Speech Communication Association; 2007 Aug. 27–31; Antwerp (Belgium). ISCA; 2007. p. 778–81. 2007. International Speech Communication Association (ISCA).

[32] Frank A, Asuncion A. UCI Machine Learning Repository [http://archive. ics. uci. edu/ml]. Irvine, CA: University of California. School of Information and Computer Science 2010;213.

[33] Jolliffe I. Principal component analysis. John Wiley & Sons, Ltd; 2002.

[34] Dhillon IS, Guan Y, Kulis B. Kernel k-means: spectral clustering and normalized cuts. In Proceedings of the tenth ACM SIGKDD international conference on Knowledge discovery and data mining 2004 Aug 22 (pp. 551–556). ACM.

[35] Shannon CE. A mathematical theory of communication. ACM SIGMOBILE Mobile Computing and Communications Review. 2001 1 1;5(1):3–55.

[36] Kwak N, Choi CH. Input feature selection by mutual information based on Parzen window. IEEE Transactions on Pattern Analysis and Machine Intelligence. 2002 12;24(12):1667–71.

[37] Peng H, Long F, Ding C. Feature selection based on mutual information criteria of max-dependency, max-relevance, and min-redundancy. IEEE Transactions on pattern analysis and machine intelligence. 2005 8;27(8):1226–38. doi: 10.1109/TPAMI.2005.159 16119262 10.1109/TPAMI.2005.159

[38] Sakar CO, Kursun O. A method for combining mutual information and canonical correlation analysis: predictive mutual information and its use in feature selection. Expert Systems with Applications. 2012 2 15;39(3):3333–44.

[39] Hsu CW, Lin CJ. A comparison of methods for multiclass support vector machines. IEEE transactions on Neural Networks. 2002 3;13(2):415–25. doi: 10.1109/72.991427 18244442 10.1109/72.991427

[40] Akbani R, Kwek S, Japkowicz N. Applying support vector machines to imbalanced datasets. In European conference on machine learning 2004 Sep 20 (pp. 39–50). Springer Berlin Heidelberg.

[41] Chawla NV. Data mining for imbalanced datasets: An overview In Data mining and knowledge discovery handbook 2005 (pp. 853–867). Springer US.

[42] Dietterich TG. Ensemble methods in machine learning In International workshop on multiple classifier systems 2000 6 21 (pp. 1–15). Springer Berlin Heidelberg.

[43] Alpaydin E. Introduction to machine learning. MIT press; 2014 8 22.

[44] Tsanas A, Little MA, McSharry PE, Spielman J, Ramig LO. Novel speech signal processing algorithms for high-accuracy classification of Parkinson's disease. IEEE Transactions on Biomedical Engineering. 2012 5;59(5):1264–71. doi: 10.1109/TBME.2012.2183367 22249592 10.1109/TBME.2012.2183367

[45] Lu L, Zhang HJ, Li SZ. Content-based audio classification and segmentation by using support vector machines. Multimedia systems. 2003 4 1;8(6):482–92.

[46] Lin CC, Chen SH, Truong TK, Chang Y. Audio classification and categorization based on wavelets and support vector machine. IEEE Transactions on Speech and Audio Processing. 2005 9;13(5):644–51.

[47] Amami R, Ayed DB, Ellouze N. An empirical comparison of SVM and some supervised learning algorithms for vowel recognition. arXiv preprint arXiv:1507.06021. 2015 Jul 22.

[48] Lu L, Li SZ, Zhang HJ. Content-based audio segmentation using support vector machines. In Proc. ICME 2001 8 22 (Vol. 1, pp. 749–752).

